# Treatment with n-3 Polyunsaturated Fatty Acids Overcomes the Inverse Association of Vitamin D Deficiency with Inflammation in Severely Obese Patients: A Randomized Controlled Trial

**DOI:** 10.1371/journal.pone.0054634

**Published:** 2013-01-25

**Authors:** Bianca K. Itariu, Maximilian Zeyda, Lukas Leitner, Rodrig Marculescu, Thomas M. Stulnig

**Affiliations:** 1 Clinical Division of Endocrinology and Metabolism, Department of Internal Medicine III, Medical University of Vienna, Vienna, Austria; 2 Christian Doppler Laboratory for Cardio-Metabolic Immunotherapy, Medical University of Vienna, Vienna, Austria; 3 Department of Laboratory Medicine, Medical University of Vienna, Vienna, Austria; Brigham & Women's Hospital, and Harvard Medical School, United States of America

## Abstract

Obesity affects the vitamin D status in humans. Vitamin D and long-chain n-3 polyunsaturated fatty acids (PUFA) provide benefit for the prevention of fractures and cardiovascular events, respectively, and both are involved in controlling inflammatory and immune responses. However, published epidemiological data suggest a potential interference of n-3 PUFA supplementation with vitamin D status. Therefore, we aimed to investigate in a randomized controlled clinical trial whether treatment with long chain n-3 PUFA affects vitamin D status in severely obese patients and potential interrelations of vitamin D and PUFA treatment with inflammatory parameters. Fifty-four severely obese (BMI≥40 kg/m2) non-diabetic patients were treated for eight weeks with either 3.36 g/d EPA and DHA or the same amount of butter fat as control. Changes in serum 25-hydroxy-vitamin D [25(OH)D] concentrations, plasma fatty acid profiles and circulating inflammatory marker concentrations from baseline to end of treatment were assessed. At baseline 43/54 patients were vitamin D deficient (serum 25(OH)D concentration <50 nmol/l). Treatment with n-3 PUFA did not affect vitamin D status (*P* = 0.91). Serum 25(OH)D concentration correlated negatively with both IL-6 (*P* = 0.02) and hsCRP serum concentration (*P* = 0.03) at baseline. Strikingly, the negative correlations of 25(OH)D with IL-6 and hsCRP were lost after n-3 PUFA treatment. In conclusion, vitamin D status of severely obese patients remained unaffected by n-3 PUFA treatment. However, abrogation of the inverse association of 25(OH)D concentration with inflammatory markers indicated that n-3 PUFA treatment could compensate for some detrimental consequences of vitamin D deficiency.

**Trial Registration:**

ClinicalTrials.gov NCT00760760

## Introduction

Vitamin D is a pleiotropic prohormone [1], which regulates calcium metabolism and helps to preserve bone mass and prevent fractures [2]. On the other hand, vitamin D is also involved in immunological processes [3]. In humans, the vitamin D status is determined by quantifying the concentration of 25-hydroxyvitamin D in serum [25(OH)D] [1]. Recommended levels, i.e. serum 25(OH)D concentration >50 nmol/l [4] are rarely achieved in children and adults [5], [6], particularly in obese subjects [7]. Vitamin D binding protein (VDBP) is a glycoprotein involved in the transport and preservation of vitamin D and alterations in its circulating concentration affect the availability and function of 25(OH)D [8]. The risk of developing obesity-related complications such as insulin resistance and type 2 diabetes is proportional to the degree of obesity [9] and tightly correlated with chronic low-grade adipose and systemic inflammation [10], [11] as well as vitamin D deficiency [12]. In addition to insulin resistance and type 2 diabetes, obesity is independently associated with a greater risk of hypertension, stroke, atherosclerotic, cardiovascular and neurodegenerative disease, cancer and death [13], [14]. Vitamin D deficiency is associated with hypertension, atherosclerosis, increased risk of myocardial infarction, cognitive decline, some types of cancer and overall increased mortality risk [15], [16], [17], [18], [19]. Consistent evidence from large well designed trials on the effect of vitamin D supplementation and prevention of the above mentioned obesity associated complications is currently lacking. However, long chain n-3 polyunsaturated fatty acids (PUFA) are known for their anti-inflammatory and cardio-protective effects [20], [21], which renders them as promising option for prevention of obesity-associated cardio-metabolic complications.

Recently, epidemiological data indicated that vitamin D status of elderly patients receiving vitamin D supplementation was negatively affected by PUFA ingestion [22]. Considering the paucity of data available on the possible interference between long chain n-3 PUFA and vitamin D, we aimed to investigate the impact of a high dose n-3 PUFA treatment on vitamin D status and a possible interaction with the anti-inflammatory effects of vitamin D in severely obese subjects.

## Subjects and Methods

The protocol for this trial and supporting CONSORT checklist are available as supporting information; see Checklist S1 and Protocol S1.

### Ethics Statement

The study was performed in compliance with the Declaration of Helsinki and Good Clinical Practice guidelines and has been approved by the Ethics Committee of the Medical University of Vienna (EK-Nr. 488/2006). All participants provided written informed consent. The trial was registered at clinicaltrials.gov with the identification no. NCT00760760.

### Subjects

**Figure 1:**
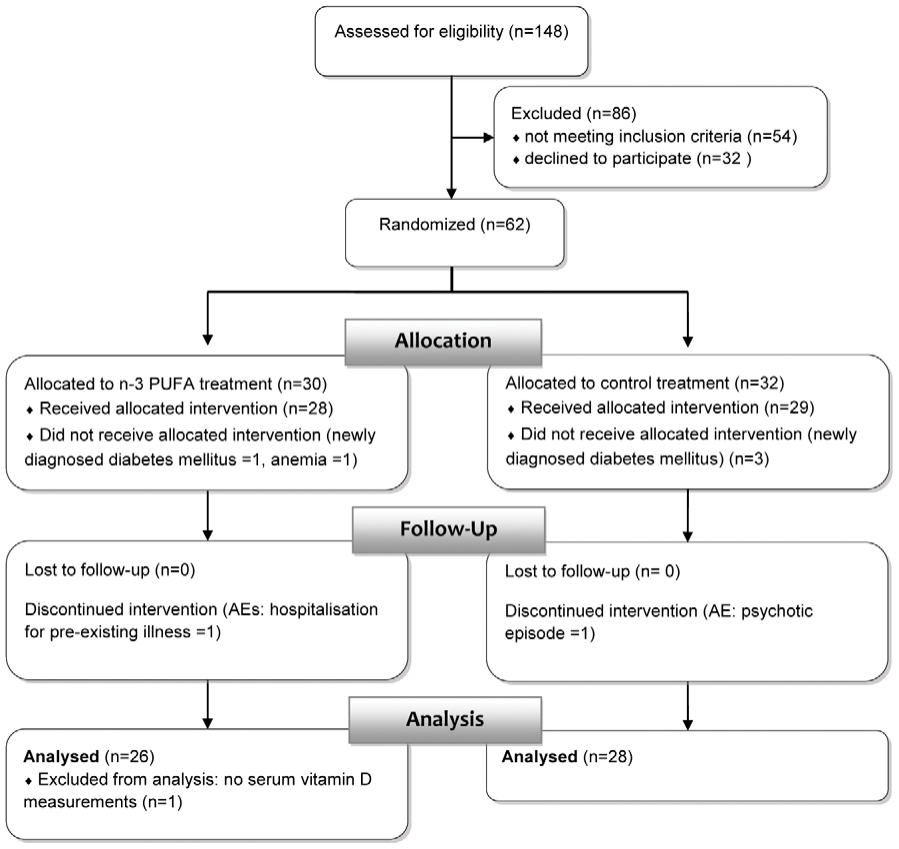
CONSORT flowchart, adapted from [21].

Fifty-five severely obese (BMI≥40 kg/m^2^), non-diabetic (fasting plasma glucose <126 mg/dl and 2 hr plasma glucose after a 75 g oral glucose tolerance test <200 mg/dl) patients were enrolled and completed an open randomized controlled clinical trial between August 2008 and July 2010. The trial has been described in detail elsewhere [21]. One patient was excluded because of a lack of serum 25(OH)D measurements at both time-points (Figure 1). Patients were randomized to receive either a 3.36 g long chain n-3 PUFA (4 capsules/d Omacor®, Solvay Pharma, Austria, each containing 460 mg eicosapentaenoic acid (EPA), 380 mg docosahexaenoic acid (DHA) and tocopherol as anti-oxidant) or an isocaloric amount of butterfat as a control for 8 weeks. At baseline and at the end of treatment anthropometric measurements (BMI, hip, waist) were performed and concentration of serum 25(OH)D, parathyroid hormone (PTH), systemic inflammatory markers and metabolic parameters as well as plasma fatty acid profiles were assessed.

### Laboratory analysis

Serum 25(OH)D, PTH and VDBP concentrations were measured in serum samples obtained from all patients both at baseline and at the end of the treatment. Serum 25(OH)D concentration was analyzed by chemiluminescent immunoassay (CLIA - Liaison®, DiaSorin, Italy) with interassay coefficients of variation of 7–9% and the lowest reportable value at 10 nmol/l. Serum PTH concentration was measured by electrochemiluminescence immunoassay (Elecsys® PTH (7–84), Roche, Basel, Switzerland) with interassay coefficients of variation of 6–8% and the lowest reportable value at 1 pg/ml. Serum VDBP concentration was measured by enzyme linked immunosorbend assay (R&D Systems, Techne Corporation, Minneapolis, USA) with interassay coefficients of variation of 5.1–7.4% and the minimum detectable dose ranged from 0.15–3.74 ng/ml. The normal range for serum samples from apparently healthy volunteers is 55.9–473 µg/ml, according to the product datasheet. We used commercial enzyme-linked immunosorbent assays (R&D Systems, Techne Corporation, Minneapolis, USA) to measure plasma concentration of interleukin (IL)-6 and serum concentration of high sensitivity C-reactive protein (hsCRP). Plasma phospholipids were isolated by thin layer chromatography and fatty acids profiles were determined by gas chromatography and electron-impact ionization mass spectroscopy as described by us in detail elsewhere [21]. Fatty acids were expressed as mol% of total fatty acids. We calculated the sum of all detected monounsaturated fatty acids (MUFA) as ∑(16:1, 18:1, 20:1, 24:1), the sum of all PUFA as ∑[18:2(n-6), 18:3(n-3), 18:3(n-6), 20:2(n-6), 20:3(n-6), 20:4(n-6), 20:5(n-3), 22:4(n-6), 22:5(n-3), 22:6(n-6)], the sum of all n-3 PUFA as ∑[18:3(n-3), 20:5(n-3), 22:5(n-3), 22:6(n-3)] and the sum of all n-6 PUFA as ∑[18:2(n-6), 18:3(n-6), 20:2(n-6), 20:3(n-6), 20:4(n-6), 22:4(n-6)].

### Statistical analysis

Statistical analysis included all patients who completed the trial and had detectable serum vitamin D levels (n = 54). Normally distributed data are presented as mean ± SEM, otherwise as median (IQR). Baseline differences between the n-3 PUFA and the control group as well as the vitamin D deficient and vitamin D non-deficient group were calculated by analysis of variance (ANOVA) for normally distributed data, otherwise by Mann-Whitney-U test. In order to compare treatment effects on vitamin D status, VDBP and PTH levels we used a one-way between-groups analysis of covariance (ANCOVA) [23]. The independent variable was the treatment group and the dependent variables were the serum 25(OH)D, VDBP and PTH concentrations at the end of the treatment, adjusted for the respective baseline values as covariates. Correlations were explored by Spearman's rank method. As recommended, sample size was calculated *a priori* for the primary end-point of the original study, ie inflammatory gene expression in adipose tissue. Sample size was thus calculated to 25 per group in order to detect a 50% change in expression of inflammatory genes at a two-sided alpha level of 0.05 with a power of 80% [21]. All analyses were performed with PASW Statistics 18 (SPSS Inc., IBM Corporation, New York, USA). Differences were considered statistically significant at two sided values of *P*<0.05.

## Results

Baseline characteristics of the study participants according to their respective treatment allocation (n-3 PUFA, control) were described elsewhere [21]. We showed that treatment with n-3 PUFA significantly reduced circulating IL-6 concentration, without affecting the hsCRP [21]. At baseline, forty-three out of 54 patients were vitamin D deficient as defined by serum 25(OH)D concentration <50 nmol/l. At baseline, mean serum 25(OH)D concentration was similar in both treatment groups (33.3±2.7 nmol/l and 39.8±4.0 nmol/l, in the n-3 PUFA and control group, respectively; *P* = 0.19), but much lower than the recommended range of 75–150 nmol/l [4]. Mean baseline PTH serum concentrations were in the normal range and not significantly different between the groups (40.0±2.9 pg/ml and 38.9±2.9 pg/ml, in the n-3 PUFA and control group, respectively; *P* = 0.80) at randomization and there was no association between serum 25(OH)D and serum PTH concentrations. VDBP serum concentration in this severely obese cohort was in the normal range and did not differ between the n-3 PUFA and control group at baseline (253.4±18.9 µg/ml and 264.5±15 µg/ml, respectively, *P* = 0.77). There was no association between serum VDBP, 25(OH) and PTH concentration.

**Figure 2:**
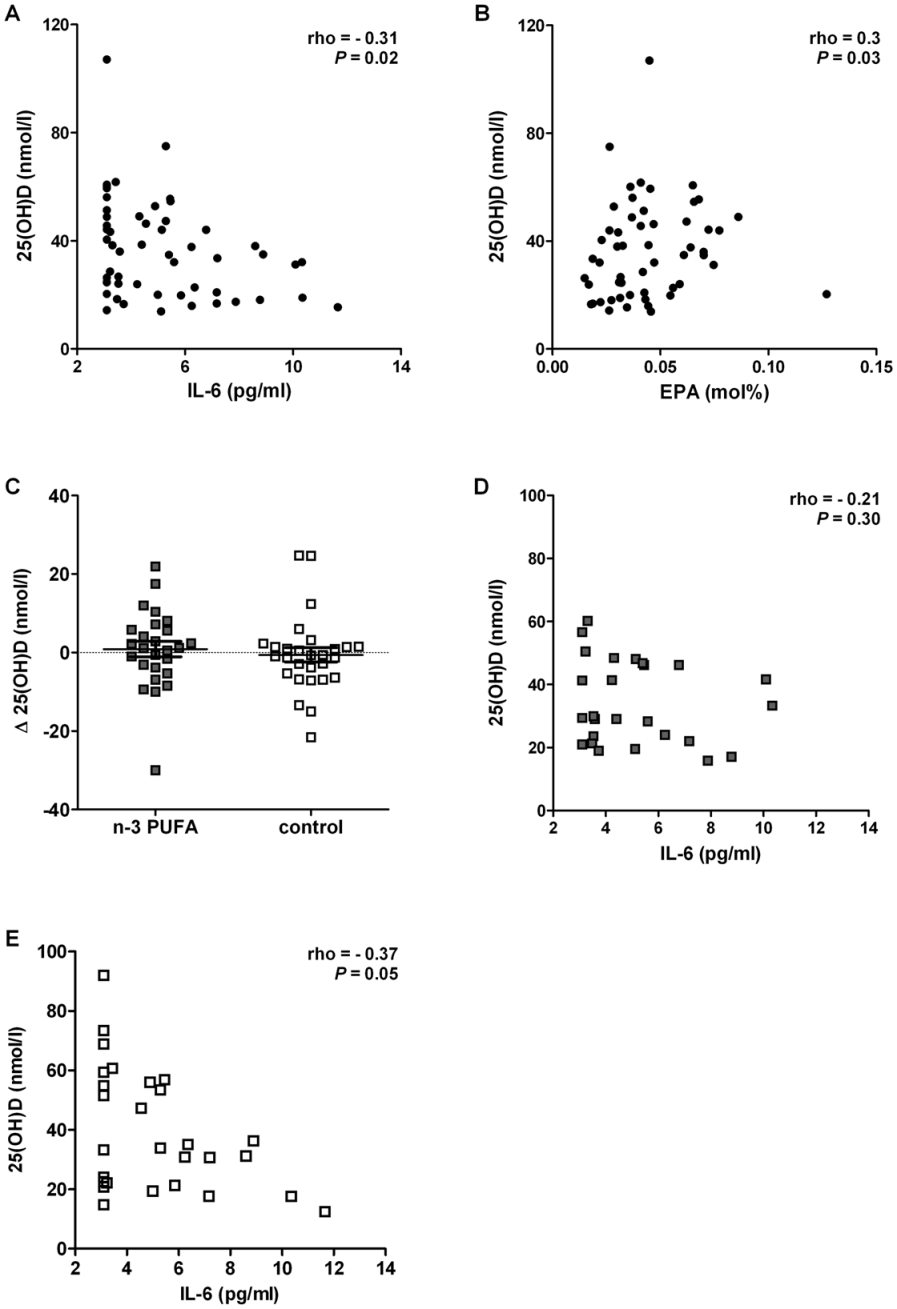
Vitamin D, n-3 PUFA and inflammation. A, B. Correlations of serum 25(OH)D concentrations with IL-6 and EPA in severely obese patients at baseline. Serum 25(OH)D concentrations of obese patients (n = 54), plotted against (A) plasma IL-6 concentration and (B) eicosapentaenoic acid (EPA) in plasma phospholipids at baseline. Statistical analysis was performed by Spearman's rank correlation test. C. The effect of long chain n-3 PUFA treatment on serum 25(OH)D concentrations. The difference (Δ) between serum 25(OH)D concentration at the end of treatment vs. its baseline value in both n-3 PUFA treated patients (n = 26) and controls (n = 28) was not statistically significant (*P* = 0.58 in ANOVA). D, E. Correlation of serum 25(OH)D concentrations with IL-6 in severely obese n-3 PUFA and control treated patients at study end. Serum 25(OH)D concentration of (D) n-3 PUFA treated patients (n = 26) and (E) controls (n = 28) plotted against plasma IL-6 concentration at the end of the intervention. Statistical analysis was performed by Spearman's rank correlation.

Patients with and without vitamin D deficiency were compared with respect to anthropometric (BMI) and inflammatory parameters, as well as fatty acid content of plasma phospholipids (Table 1). Vitamin D deficient patients were younger and had higher circulating IL-6 concentrations than their non-deficient counterparts (both *P* = 0.04). In addition, baseline serum 25(OH)D concentration negatively correlated with BMI (Spearman's rho = −0.33, *P* = 0.01) as well as plasma inflammatory markers IL-6 (Spearman's rho = −0.31, *P* = 0.02; Figure 2A) and hsCRP (Spearman's rho = −0.29, *P* = 0.03), in the whole group. The negative association between serum 25(OH)D and plasma IL-6 concentration remained significant even after adjusting for age (r = −0.31, *P* = 0.02). On the contrary, baseline 25(OH)D positively correlated with relative plasma EPA concentration (Spearman's rho = 0.3, *P* = 0.03; Figure 2B) and total n-3 PUFA (Spearman's rho = 0.27, *P* = 0.048) in the whole study population, but not with total PUFA, total n-6 PUFA and total MUFA concentrations (not shown).

**Table 1:** Characteristics of vitamin D-deficient and non-deficient study subjects at baseline.

	25(OH)D≤50 nmol/l	25(OH)D>50 nmol/l
	(n = 43)	(n = 11)
Group (n-3 PUFA/control)	22/21	4/7
Sex (f/m)	37/6	9/2
Age (y)^1^	37±2	45±4*
BMI (kg/m2)	46.6 (43.1, 50.8)	42.7 (41.1, 50.7)
Interleukin-6 (ng/ml)	4.7 (3.5, 8.3)	3.7 (3.1, 4.3)*
hsCRP (mg/dl)	0.78 (0.40, 1.67)	0.59 (0.27, 0.86)
PTH (pg/ml)	40.7±2.4	34.5±2.3
Calcium (mmol/L)	2.4±0.01	2.3±0.02
VDBP (µg/ml)	256.2±13.0	272.3±29.4
EPA (mol%)	0.04±0.00	0.05±0.00
DHA (mol%)	0.25±0.01	0.24±0.02
Total n-3 PUFA (mol%)	0.43±0.02	0.44±0.03
Total n-6 PUFA (mol%)	7.3±0.1	7.4±0.2
Total PUFA (mol%)	7.8±0.1	7.9±0.2
Total MUFA (mol %)	2.8±0.1	2.6±0.1

1 Data presented as mean ± SEM for normally distributed data, otherwise median (IQR). No statistical significant differences between the analyzed parameters in vitamin D deficient and non-deficient patients were found, except for age plasma interleukin-6 concentration, indicated by asterisk.

* (both *P* = 0.04, calculated by ANOVA and Mann-Whitney-U Test).

BMI, body mass index; hsCRP, high sensitive C-reactive protein; PTH, parathyroid hormone; EPA, eicosapentaenoic acid; DHA, docosahexaenoic acid; MUFA, sum of all detected monounsaturated fatty acids; VDBP, vitamin D binding protein.

Treatment with long chain n-3 PUFA and control did not impact serum 25(OH)D concentration (34.2±2.6 nmol/l and 39.2±3.8 nmol/l in the n-3 PUFA and control group at treatment end, respectively, *P* = 0.91; Figure 2C), serum PTH concentration (41.7±3.1 pg/ml and 40.8±3.0 pg/ml in the n-3 PUFA and control group at treatment end, *P* = 0.92) or serum VDBP concentration (244.9±14.3 µg/ml and 249±14.3 µg/ml in the n-3 PUFA and control group at the end of treatment, *P* = 0.95). We further determined the seasonal impact on 25(OH)D serum concentration at baseline and at the end of treatment in the whole study population and in the two groups, separately. Baseline serum 25(OH)D concentration was slightly lower in patients randomized in winter (from September to March) compared to those randomized in summer (from May to August), i.e.34.9±3.0 nmol/l vs. 41.4±4.1 nmol/l, *P* = 0.24), the difference in concentration did not reach statistical significance. Similar findings were noted at the end of the treatment (not shown).

The correlations of serum 25(OH)D with IL-6 (Spearman's rho = −0.21, *P* = 0.30; Figure 2D), total n-3 PUFA (Spearman's rho = 0.04, *P* = 0.82) and EPA (Spearman's rho = 0.13, *P* = 0.51) were lost after n-3 PUFA treatment. In contrast, the negative correlation between serum 25(OH)D and plasma IL-6 (Spearman's rho = −0.37, *P* = 0.05) and serum 25(OH)D and hsCRP (Spearman's rho = −0.41, *P* = 0.03) remained significant in the control group.

## Discussion

Vitamin D deficiency along with long chain n-3 PUFA deficiency is common in both adults and children and particularly in the obese [5]. This secondary analysis aimed to investigate whether n-3 PUFA could have deleterious effects on vitamin D status in obese individuals. Accordingly, most patients from our study population exhibited vitamin D deficiency. All patients had normal serum levels of VDBP. Under physiologic conditions, serum PTH concentrations tend to correlate negatively with serum 25(OH)D concentration in case of severe vitamin D deficiency [6], but no such correlation was found in our study, a finding which has been also reported by others [24]. There is no consensus on the threshold of 25(OH)D values below which PTH levels starts to increase. Furthermore it seems that the inverse relationship between serum PTH and 25(OH)D is not causative, but reflects biochemical abnormalities associated to obesity, as multiple linear regression analyses from other studies have demonstrated that BMI itself accounts for the decreased serum vitamin D and increased PTH concentration [24], [25].

Vitamin D and n-3 PUFA are found together in cod liver oil, known for its unpleasant taste. Since purified re-esterified n-3 PUFA have become available, cod liver oil supplementation has further lost in appeal. Thus, concerns have arisen that supplementation with n-3 PUFA derived from fish-oil might, as a side effect, cause a diminished supplementation with vitamin D. There is scarce evidence from animal models receiving combined therapy with n-3 PUFA and vitamin D, so it is difficult to speculate on their combined effect. A large trial investigating the concomitant effect of n-3 PUFA and vitamin D supplementation on the prevention of cancer and cardio-vascular disease is set to be finished by 2017 [26]. In addition, Niramitmahapanya *et al.* found that dietary PUFA intake negatively correlated with the vitamin D status of elderly patients, receiving supplemental vitamin D_3_ and suggested that n-3 PUFA treatment affects vitamin D absorption [22]. We could show here that a considerable daily n-3 PUFA dose of 3.36 g did not impact the overall vitamin D status, irrespective of the season. VDBP concentration was also unaffected by the treatment. However, we cannot rule out that a higher n-3 PUFA dose affects vitamin D status. Inuits who consume high doses of n-3 PUFA and vitamin D from free-living fish and sea mammals have high circulating vitamin D concentrations [27]. In a cross-sectional analysis where serum 25(OH)D concentration and dietary intake of n-3 PUFA was assessed, the group with the highest vitamin D concentration had also the higher n-3 PUFA intake (1.5 g/d) [28]. These epidemiological data further argue against a significant impact of n-3 PUFA on vitamin D status. Moreover, baseline vitamin D concentrations in the obese middle aged cohort investigated here positively correlated with plasma phospholipid EPA and total n-3 PUFA content, strongly arguing against a negative effect of n-3 PUFA in the regular diet on vitamin D uptake. The duration of our study can be considered long enough to detect an interaction between n-3 PUFA and vitamin D as the half-life of vitamin D is reported at circa 1 month in humans and serum 25(OH)D is essentially at the plateau concentration by 1 month [29]. It is hence rather unlikely that a longer duration of treatment would significantly impact vitamin D status. Another factor which could clearly influence a possible interaction between n-3 PUFA and vitamin D is the type of patient. In this case, pathologies affecting vitamin D absorption and metabolism, such as intestinal, severe liver or kidney disease have to be considered. However, the existence of these conditions was excluded in our study.

Inflammation considerably contributes to obesity-associated complications such as insulin resistance, type 2 diabetes and cardiovascular disease [11]. In this regard, anti-inflammatory therapies are currently under investigation as novel preventive and therapeutic strategies. We have shown that long chain n-3 PUFA reduce the concentration of inflammatory markers such as IL-6 in severely obese subjects [21]. IL-6 regulates hsCRP production in the liver, thus a reduction in hsCRP concentration might also occur with longer treatment. Vitamin D is negatively associated with both BMI and inflammatory markers, such as IL-6 and hsCRP in healthy lean and obese subjects [30], [31]. We and others have shown that n-3 PUFA reduce systemic and adipose tissue inflammation, induce anti-inflammatory gene expression in circulating mononuclear cells and improve metabolic control in severely obese, overweight and elderly subjects [21], [32], [33]. The data presented here indicate that the anti-inflammatory action of n-3 PUFA occurs even in vitamin D deficiency. Particularly noteworthy is the finding, that n-3 PUFA but not control treatment abolished the negative association of vitamin D concentration and inflammatory parameters. Hence it could be speculated that the anti-inflammatory action of n-3 PUFA may in part compensate for the detrimental outcome of vitamin D deficiency. Clearly, causalities of these interrelations need to be investigated in future studies.

In conclusion, treatment with n-3 PUFA does not compromise the overall vitamin D status of obese patients, many of whom were vitamin D deficient, but abrogates the inverse association of vitamin D deficiency with inflammation. The fact that n-3 PUFA treatment reduces inflammation indicates that adequate n-3 PUFA intake could compensate for some detrimental outcomes of vitamin D deficiency. Cumulative effects of n-3 PUFA and vitamin D on unfavorable obesity-related complications remain to be evaluated.

## Supporting Information

Checklist S1 **CONSORT checklist.**
(DOC)

Protocol S1 **Study protocol.**
(DOC)
