# Supplementary material for: Treatment with n-3 Polyunsaturated Fatty Acids Overcomes the Inverse Association of Vitamin D Deficiency with Inflammation in Severely Obese Patients: A Randomized Controlled Trial
Source: PLoS One. 2013 Jan 25;8(1):e54634. doi: 10.1371/journal.pone.0054634 (PMC3556046; doi:10.1371/journal.pone.0054634)
Supplement: Protocol S1 — Study protocol. (DOC) [file pone.0054634.s002.doc]

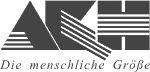

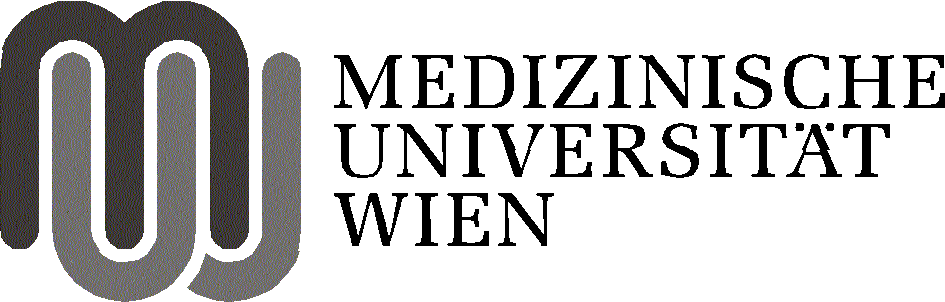


ALLGEMEINES

KRANKENHAUS

DER STADT WIEN

Währinger Gürtel 18-20 • A-1090 Wien • Ebene 6J

Tel.: (+43 1) 40400/4310 • Fax: (+43 1) 405 93 234

e-mail: anton.luger@meduniwien.ac.at

DVR: 0660167

universitätsklinik für Innere medizin iii

KLINISCHE Abteilung Für endokrinologie & stoffwechsel

Leiter: Univ.Prof.Dr.A.Luger

**Bianca Itariu, MD**

**Prof. Thomas M. Stulnig, MD**

**Study protocol**

**THE EFFECTS OF N-3 PUFA SUPPLEMENTATION ON 25-HYDROXYVITAMIN D3 STATUS IN SEVERELY OBESE PATIENTS**

**Abstract**

The aim of this study is to assess the effects of long chain n-3 polyunsaturated fatty acids (n-3 PUFA) supplementation on vitamin D status of severely obese subjects, in a randomized controlled clinical study. Vitamin D regulates a multitude of biological processes. Its deficiency is commonly observed in obesity. Moreover, broad published evidence links vitamin D deficiency to obesity-associated co-morbidities like type two diabetes mellitus or cardiovascular disease. Recently it has been shown that vitamin D absorption is influenced by the type of dietary fat consumed. Specifically increased dietary intake of polyunsaturated fatty acids was negatively associated with 25-hydroxyvitamin D in plasma from older overweight subjects. However, no data is available on n-3 PUFA supplementation and circulating vitamin D concentrations in obese subjects.

For this analysis we will determine serum 25-hydroxyvitamin D concentrations in severely obese patients from a randomized controlled clinical trial designed to determine the effect of a high dose long chain n-3 PUFA treatment on adipose and systemic inflammation. Serum 25-hydroxyvitamin D concentration will be determined at baseline and after the intervention in all patients. Considering the focus on both vitamin D and long chain n-3 PUFA treatment in prevention of obesity related complications like cardiovascular disease, we expect that our findings will shed light on possible interactions between these substances and thus contribute to enhance the knowledge in this field.

**Introduction**

Vitamin D is a pleiotropic hormone which plays a key role in many biological processes, from bone metabolism to immunological regulation and cardiovascular disease prevention (1). In humans, the vitamin D status is determined by quantifying the amount of 25-hydroxyvitamin D in serum. Although there is no consensus on the threshold for vitamin D deficiency or insufficiency, according to most international guidelines, 25-hydrohyvitamin D concentrations under 30 nmol/l are considered severely deficient, whereas a concentration of less than 50 nmol/l is also used to define vitamin D deficiency. In Austria the recommended concentration is between 75 and 125 nmol/l (Citation KIMCL website). It is estimated that approximately 1 billion people worldwide have vitamin D deficiency or insufficiency (2). Obese individuals are particularly at risk for vitamin D deficiency, presumably because of decreased bioavailability (3), (4). Moreover, obese subjects are at high risk for metabolic and cardiovascular morbidity. The risk of developing obesity-related complications is proportional to the degree of obesity and tightly correlated with chronic low-grade adipose and systemic inflammation (5) as well as vitamin D deficiency (4). Long chain polyunsaturated fatty acids of the n-3 series (n-3 PUFA) exert anti-inflammatory and cardio-protective effects (6), (Itariu et al.-manuscript in preparation). These effects render n-3 PUFA treatment of obesity-related chronic complications as very promising. Recently, it could be shown that dietary fat composition could alter the vitamin D status in elderly patients receiving supplemental vitamin D3 (7). Interestingly, the vitamin D status of these subjects correlated negatively with the calculated amount of ingested polyunsaturated fatty acids (PUFA). However, as the fat intake had been only estimated by using food frequency questionnaires, the authors could make no correlations between serum 25-hydrohyvitamin D concentrations and actual circulating fatty acid profiles of this population. Furthermore, PUFA of the n-3 and n-6 series where not analysed separately.

Here we aim to investigate whether treatment with a high dose of long chain n-3 PUFA (eicosapentaenoic acid - EPA, docosahexaenoic acid - DHA) would impact vitamin D status in severely obese non-diabetic patients. To our knowledge the role of vitamin D and marine n-3 PUFA in primary prevention of cancer and cardiovascular disease is investigated in a large ongoing clinical trial (8). Since both n-3 PUFA and vitamin D play a role in preventing cardio-vascular disease, it is of great importance that any interaction between these agents is tested rigorously.

**Study population**

The subjects of this analysis comprise all fifty-five severely obese (BMI>40 kg/m^2^) non-diabetic (fasting plasma glucose < 126 mg/dl and 2 hr plasma glucose after a 75 g OGTT < 200 mg/dl) patients from a randomized controlled clinical trial. Exclusion criteria included acute illness within the last two weeks, known diabetes mellitus or use of antidiabetic medication, acquired immunodeficiency (HIV infection, AIDS), hepatitis or other significant liver disease, severe or untreated cardiovascular, renal, pulmonary disease, untreated or inadequately treated clinically significant thyroid disease, anemia, active malignant disease, inborn or acquired bleeding disorder including Warfarin treatment, pregnancy or breast feeding, documented intolerability to n-3 PUFA. Patients were treated with either 3.3 g/d n-3 PUFA (EPA and DHA) or the same amount of butter fat as control, for eight weeks. At baseline and at the end of the intervention we performed anthropometric measurements (BMI, hip, waist, systolic and diastolic blood pressure) and blood sampling to evaluate systemic inflammatory markers and metabolic parameters as well as to determine fatty acid profiles in plasma phospholipids by thin layer chromatography followed by gas chromatography/mass spectrometry. The study was performed in compliance with the Declaration of Helsinki and Good Clinical Practice guidelines and had been approved by the Ethics Committee of the Medical University of Vienna (EK-Nr. 488/2006). We registered the study under the clinicaltrials.gov identification no. NCT00760760.

**Serum 25-hydroxyvitamin D measurements**

Serum 25-hydrohyvitamin D concentration will be measured in serum samples obtained from all patients both at baseline and at the end of the treatment. All samples have been stored at -20°C. Measurements will be made at the Department of Laboratory Medicine of the Medical University of Vienna.

**Statistical analysis**

Data will be analysed on intention-to-treat-basis. Continuous variables determined before and after the treatment will be analysed by RM-ANOVA, in a full factorial design using PASW Statistics 18 (SPSS Inc., IBM Corporation, New York, USA). Correlations will be calculated with Pearson’s method and validated by Spearman’s method. Statistically significant differences will be considered significant at p<0,05.

**Funding**

This trial was supported by the Austrian National Bank Project no. 12735 and the European Community’s 7th Framework Programme (FP7/2007-2013) under grant agreement no. 201608 (both to Prof. Thomas Stulnig). No supplementary costs should arise, as the project is part of “Amtsforschung” at the Medical University of Vienna.

**Refferences**

1. Verstuyf A, Carmeliet G, Bouillon R, Mathieu C. Vitamin D: a pleiotropic hormone. Kidney Int;78:140-5.

2. Holick MF. Vitamin D deficiency. N Engl J Med 2007;357:266-81.

3. Harris SS, Dawson-Hughes B. Reduced sun exposure does not explain the inverse association of 25-hydroxyvitamin D with percent body fat in older adults. J Clin Endocrinol Metab 2007;92:3155-7.

4. Cheng S, Massaro JM, Fox CS, et al. Adiposity, cardiometabolic risk, and vitamin D status: the Framingham Heart Study. Diabetes 2010;59:242-8.

5. Despres JP, Lemieux I, Bergeron J, et al. Abdominal obesity and the metabolic syndrome: contribution to global cardiometabolic risk. Arterioscler Thromb Vasc Biol 2008;28:1039-49.

6. Browning LM. n-3 Polyunsaturated fatty acids, inflammation and obesity-related disease. Proc Nutr Soc 2003;62:447-53.

7. Niramitmahapanya S, Harris SS, Dawson-Hughes B. Type of dietary fat is associated with the 25-hydroxyvitamin D3 increment in response to vitamin D supplementation. J Clin Endocrinol Metab 2011;96:3170-4.

8. Manson JE, Bassuk SS, Lee IM, et al. The VITamin D and OmegA-3 TriaL (VITAL): Rationale and design of a large randomized controlled trial of vitamin D and marine omega-3 fatty acid supplements for the primary prevention of cancer and cardiovascular disease. Contemp Clin Trials 2011.
